# The Impact of Workplace-Based Violence on Resident Doctor Wellbeing and Retention and How to Improve Current Guidelines and Support

**DOI:** 10.1192/bjo.2026.11499

**Published:** 2026-06-30

**Authors:** Win Min Thet, Sukh Bahia, Rowena Carter, Layth Humsi

**Affiliations:** Central and Northwest London NHS Trust, London, United Kingdom

## Abstract

**Aims:**

The aim of the project is to explore resident doctors’ experience of workplace-based violence within Central and Northwest London NHS Foundation Trust. These findings will help:

1) To identify barriers to reporting incidents.

2) To improve access to wellbeing support provided to resident doctors.

3) To update serious incident policy for resident doctors.

**Methods:**

Both qualitative and quantitative surveys were conducted among all the resident doctors in Central and Northwest London NHS Foundation Trust.

Quantitative Survey: Between January and February 2025, Resident Doctors within Central and Northwest London NHS Trust were invited to complete a 13-item quantitative survey. 34 responses were received.

Qualitative Interviews: Following responses from quantitative surveys, seven resident doctors who reported workplace-based violence through Datix agreed to participate in semi-structured qualitative interviews, consisting of 13 questions. All the interviews were conducted through Microsoft teams and transcribed. Interview responses were analysed through thematic analysis to identify common themes and subthemes of individual perception and reflection on their experience of violence.

**Results:**

Quantitative Survey:

34 resident doctors from different grades (foundation year doctors, GP trainees, core trainees, and higher trainees) across five different boroughs responded to quantitative surveys. Of these, five core trainees, three higher trainees, two foundation year trainees and two GP trainees reported the violence.

These resident doctors who experience violence are 40% from inpatient units and 60% from community clinics. Physical and non-physical violence are most common types of violence that resident doctors experienced. Only 7 out of 20 resident doctors who experienced workplace violence reported the incident through the incident reporting system called Datix.

Qualitative Survey:

Seven resident doctors participated in interviews exploring their experience of workplace-based violence. Participants include one higher trainee, one GP trainee and five core trainees. Participants highlighted there were significant impacts of violence or aggression on emotional wellbeing as well as professionally on career commitment. A common theme emerging from the semi-structured interviews was that resident doctors who experienced physical violence were preceded by non-physical or verbal violence.

**Conclusion:**

Resident doctors experiencing workplace violence and aggression in the NHS is common across different specialities, among which unfortunately psychiatry resident doctors experience the most.

In addition, resident doctors in training do not have always access to the help and support that they need following these incidents. Quantitative survey and Qualitative thematic analysis of this project call for Improved access to various wellbeing resources and Individual support for all resident doctors who experience workplace-based violence.

